# Identification of the Additional Mitochondrial Liabilities of 2-Hydroxyflutamide When Compared With its Parent Compound, Flutamide in HepG2 Cells

**DOI:** 10.1093/toxsci/kfw126

**Published:** 2016-07-13

**Authors:** Amy L. Ball, Laleh Kamalian, Ana Alfirevic, Jonathan J. Lyon, Amy E. Chadwick

**Affiliations:** *Department of Molecular and Clinical Pharmacology, MRC Centre for Drug Safety Science, University of Liverpool, Liverpool L69 3GE, UK; ^†^Department of Molecular and Clinical Pharmacology, The Wolfson Centre for Personalised Medicine, University of Liverpool, Liverpool L69 3GL, UK; ^‡^GlaxoSmithKline, Safety Assessment, Ware SG12 0DP, UK

**Keywords:** flutamide, DILI, 2-hydroxyflutamide, mitochondria.

## Abstract

The androgen receptor antagonist, flutamide, is strongly associated with idiosyncratic drug-induced liver injury (DILI). Following administration, flutamide undergoes extensive first-pass metabolism to its primary metabolite, 2-hydroxyflutamide. Flutamide is a known mitochondrial toxicant; however there has been limited investigation into the potential mitochondrial toxicity of 2-hydroxyflutamide and its contribution to flutamide-induced liver injury. In this study we have used the acute glucose or galactose-conditioning of HepG2 cells to compare the mitochondrial toxicity of flutamide, 2-hydroxyflutamide and the structurally-related, non-hepatotoxic androgen receptor antagonist, bicalutamide. Compound-induced changes in mitochondrial oxygen consumption rate were assessed using Seahorse technology. Permeabilization of cells and delivery of specific substrates and inhibitors of the various respiratory complexes provided more detailed information on the origin of mitochondrial perturbations. These analyses were supported by assessment of downstream impacts including changes in cellular NAD^+^/NADH ratio. Bicalutamide was not found to be a mitochondrial toxicant, yet flutamide and 2-hydroxyflutamide significantly reduced basal and maximal respiration. Both flutamide and 2-hydroxyflutamide significantly reduced respiratory complex I-linked respiration, though 2-hydroxyflutamide also significantly decreased complex II and V-linked respiration; liabilities not demonstrated by the parent compound. This study has identified for the first time, the additional mitochondrial liabilities of the major metabolite, 2-hydroxyflutamide compared with its parent drug, flutamide. Given the rapid production of this metabolite upon administration of flutamide, but not bicalutamide, we propose that the additional mitochondrial toxicity of 2-hydroxyflutamide may fundamentally contribute to the idiosyncratic DILI seen in flutamide-treated, but not bicalutamide-treated patients.

Drug-induced liver injury (DILI) represents a significant burden to health organizations and is a major contributor to cases of drug attrition (Kia *et al.*, 2013). Recent figures estimate 20 new cases of DILI per 100 000 persons of the general population, with idiosyncratic DILI accounting for 11% of acute liver failure cases in the United States (Leise *et al.*, 2014; Yuan and Kaplowitz, 2013). Idiosyncratic cases of DILI, characterized by differences in individual susceptibility and a complex dose-response relationship, make it difficult to identify compounds with DILI liabilities prior to marketing (Chalasani *et al.*, 2014).

There are multiple mechanisms associated with the onset of DILI, and pre-clinical investigations are insensitive to some of them, contributing to the overall poor prediction of DILI. Possible mechanisms amongst these are the generation of reactive metabolites and mitochondrial dysfunction (Boelsterli and Lim, 2007; Russmann *et al.*, 2009). The high exposure of the liver to *in situ*-generated reactive metabolites results in this organ being particularly susceptible (Srivastava *et al.*, 2010). The ionizing conditions of the mitochondria encourage the accumulation of cationic compounds in this organelle, and this, coupled with the critical role of mitochondria in ATP production and apoptosis culminates in the potential for critical cellular injury upon mitochondrial dysfunction (Begriche *et al.*, 2011; Pessayre *et al.*, 2010).

Flutamide, an androgen receptor antagonist, was first marketed for the treatment of prostate cancer in 1989. Cases of idiosyncratic DILI were subsequently reported, with cases of hospitalization or death in 0.03% of flutamide-treated patients, primarily presenting with cholestatic hepatitis (Chu *et al.*, 1998; Wysowski and Fourcroy, 1996). As a result, in 1999 the Food and Drug Administration applied a black box warning regarding the risk of hepatic necrosis and cholestasis in flutamide-treated patients (Coe *et al.*, 2007).

*In vitro* studies have shown the inhibition of mitochondrial respiratory complex I (NADH ubiquinone oxidoreductase) activity by flutamide (Coe *et al.*, 2007). However, upon administration, flutamide undergoes extensive first-pass metabolism, primarily by conversion to 2-hydroxyflutamide via cytochrome P450 1A2 (CYP1A2), followed by glucuronidation before excretion (Figure 1) (Coe *et al.*, 2007; Shet *et al.*, 1997). Following a single 250 mg dose of flutamide its maximum plasma concentration (*C*_max_) is 72.2 nM, yet the *C*_max_ of 2-hydroxyflutamide is 4.4 µM (Schulz *et al.*, 1988). Despite the longer half-life (9.6 vs 7.8 h) and higher *C*_max_ of 2-hydroxyflutamide compared with its parent compound, the potential mitochondrial toxicity of 2-hydroxyflutamide as a contributor to flutamide-induced liver injury has not been fully investigated (Kostrubsky *et al.*, 2007). We hypothesized that because patients are exposed to such a high concentration of the 2-hydroxyflutamide metabolite that it may contribute significantly to the toxicity exhibited upon flutamide administration and possibly through a mitochondrial mechanism.

**FIG. 1:**
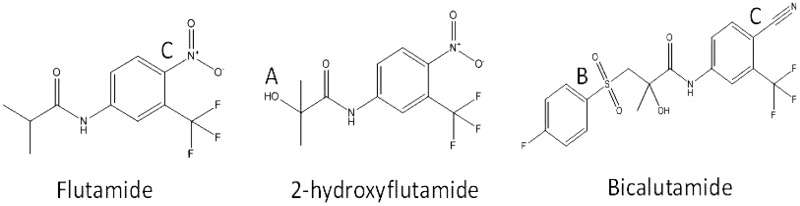
Flutamide, 2-hydroxyflutamide and bicalutamide chemical structures. Flutamide is rapidly hydroxylated **(A)** to 2-hydroxyflutamide upon administration. This primary metabolite has been shown to have higher androgen receptor binding affinity than its parent compound (Shet *et al.*, 1997). Bicalutamide was derived from flutamide by the addition of a 4-fluorophenylsulfonyl moiety **(B)** and also notably replaces the nitroaromatic structural alert in flutamide with a cyano group **(C).**

Hepatoma-derived HepG2 cells are often used as a model system for the identification of compounds with hepatic liabilities in early compound development. In an aerobic, high-glucose environment, such as in normal cell culture, these cells use oxidative phosphorylation (OXPHOS) as their main source of ATP; however, they have the ability to increase utilization of aerobic glycolysis for ATP production if required; rendering them less susceptible to mitochondrial toxicants (Diaz-Ruiz *et al.*, 2011; Marroquin *et al.*, 2007; Rodríguez‐Enríquez *et al.*, 2001). Replacing glucose in medium with galactose reduces the ATP yield from glycolysis, so increases the reliance on OXPHOS for ATP production in HepG2 cells; providing increased sensitivity to mitochondrial toxicants than in glucose-conditioned cells (Kamalian *et al.*, 2015; Marroquin, *et al.*, 2007).

This study aimed to compare the mitochondrial toxicity induced by 2-hydroxyflutamide to that induced by flutamide, initially by comparing measurements of cytotoxicity and cellular ATP content in glucose-conditioned HepG2 cells with the same endpoints in HepG2 cells subject to acute galactose-conditioning. Bicalutamide, a non-mitotoxic structural counterpart of flutamide which is not associated with idiosyncratic DILI was also tested as a negative control (Figure 1). Subsequently, oxygen consumption rate (OCR) assessment and HepG2 cell permeabilization were used for in-depth analysis of mitochondrial dysfunction, followed by assessment of the downstream impact of perturbations induced by the parent compound and metabolite.

It was demonstrated for the first time, to the best of our knowledge, that 2-hydroxyflutamide, like flutamide, is an inhibitor of respiratory complex I in HepG2 cells. In addition, 2-hydroxyflutamide was found to be an inhibitor of respiratory complexes II (succinate dehydrogenase) and V (ATP synthase) activity, perturbations not induced by the parent compound. This study has highlighted the importance of the toxicological analysis of major chemical metabolites and identifies 2-hydroxyflutamide as a potential significant contributor to flutamide-induced liver injury. These findings are not only relevant to the development of future androgen receptor antagonists but to all therapeutics in which hepatic dysfunction potentially due to mitochondrial dysfunction is evident.

## MATERIALS AND METHODS

### 

#### Materials

All forms of DMEM, mitoSOX red indicator and rat tail collagen I were purchased from Life Technologies (Paisley, UK). HepG2 cells were purchased from European Collection of Cell Cultures (ECACC, Salisbury, UK). NAD^+^/NADH ratio assay kit was purchased from eEnzyme (Maryland, USA). Cytotoxicity detection kits were purchased from Roche Diagnostics Ltd (West Sussex, UK). Clear and white 96-well plates were purchased from Fisher Scientific (Loughborough, UK) and Greiner Bio-One (Stonehouse, UK) respectively. All OCR consumables were purchased from Seahorse Bioscience (North Billerica, Massachusetts, USA). All other reagents and chemicals were purchased from Sigma Aldrich (Dorset, UK).

#### Cell Culture

HepG2 cells were maintained in DMEM high-glucose medium (glucose; 25 mM) supplemented with FBS (10% v/v), L-glutamine (4 mM), sodium pyruvate (1 mM), and HEPES (1 mM). All cells were incubated at 37 °C and 5% CO_2_. Cells were used up to passage 20.

#### Cell Plating and Metabolic Switch

Metabolic switching of cells was performed based on work previously described by (Kamalian *et al.*, 2015). Briefly, 24 h prior to metabolic switch, HepG2 cells were collected by trypsinization and seeded on a collagen-coated flat-bottomed 96-well plate (20 000 cells/50 µl/well) and incubated (37°C, 5% CO_2_) overnight. On the morning of the experiment, cells were washed 3 times in either glucose or galactose medium (DMEM containing 25 mM glucose and 4 mM L-glutamine or 10 mM galactose and 6 mM L-glutamine respectively (extra L-glutamine was provided to ensure this was not a limiting factor upon increased OXPHOS reliance), plus supplements listed in cell culture, only dialyzed FBS (to remove trace amounts of glucose) was used in galactose medium before incubation (2 h, 37**°**C, 5% CO_2_). Flutamide, 2-hydroxyflutamide and bicalutamide stock solutions were prepared in DMSO and diluted further in the appropriate medium. Compounds (50 µl) were then added to each well and cells were incubated for 2 h (37 °C, 5% CO_2_). All assays used ≤ 0.5% DMSO as a vehicle control.

#### Dual Assessment of Mitochondrial Function (ATP Content) Alongside Cytotoxicity (LDH Release)

Following compound incubation (0.05–300 µM flutamide, 2-hydroxyflutamide or bicalutamide) with cells in glucose or galactose medium (2 h), cell supernatant was extracted and the cells lysed using somatic cell ATP releasing agent. LDH content of the cells and supernatant was assessed using a cytotoxicity detection kit according to the manufacturer’s instructions. ATP content was assessed using an ATP bioluminescent assay according to the manufacturer’s instructions. Briefly, 10 µl of cell lysate or ATP standard curve solutions were added to a white-walled 96-well plate before addition of 40 µl ATP assay mix. Protein content of all wells was then assessed using a BCA assay applied to 10 µl of cell lysate and standards. LDH (490 nm), BCA (570 nm), and ATP assay luminescence were then measured using a plate reader (Varioskan, Thermo Scientific). ATP content readings were normalized to protein content.

#### OCR Analysis

##### Assay preparation

HepG2 cells were collected by trypsinization and seeded on a collagen-coated XF 96-well cell culture microplate (25 000 cells/100 µl medium/well) and incubated overnight in glucose medium (37 °C, 5% CO_2_). All assays used ≤ 0.5% DMSO as a vehicle control.

##### Mitochondrial stress test

**FIG. 2:**
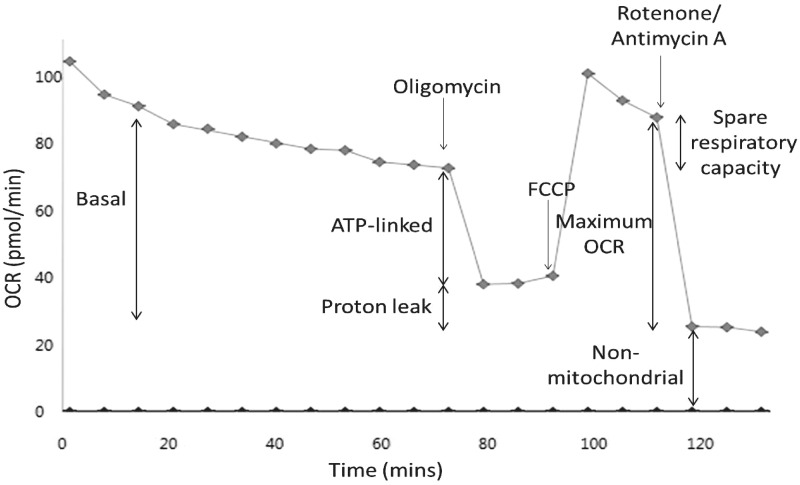
Representative control mitochondrial stress test trace. Mitochondrial stress test assays consisted of a series of compound injections into the cell culture microplate. Flutamide/2-hydroxyflutamide or vehicle control (shown) was first injected, followed by 9 measurement cycles. Remaining injections consisted of oligomycin (ATP synthase inhibitor), FCCP (OXPHOS uncoupler), and rotenone/antimycin A (complex I and III inhibitors, respectively) with each followed by 3 measurement cycles. This series of manipulations enabled the calculation of parameters: basal, ATP-linked, maximum, and non-mitochondrial OCR, as well as proton leak. Each measurement cycle was a total of 6 min.

Cells were incubated for 1 h (37**°**C, 0% CO_2_) before culture medium was replaced by 175 µl of unbuffered Seahorse XF Base medium supplemented with glucose (25 mM), L-glutamine (2 mM), sodium pyruvate (1 mM), pre-warmed to 37 °C (pH 7.4). Prior to measurement of OCR, the Seahorse XFe96 instrument gently mixed the assay medium in each well for 10 min to enable the oxygen partial pressure to reach equilibrium. The OCR was then measured 3 times to establish a baseline rate prior to the acute injection of flutamide, 2-hydroxyflutamide or bicalutamide (7.8–500 µM). There were 9 OCR measurement cycles following compound injection and each measurement cycle consisted of a 3 min mix and 3 min measure. Following this compound incubation (54 min), a mitochondrial stress test was performed consisting of sequential injections of oligomycin (1 µM), carbonyl cyanide 4-(trifluoromethoxy) phenylhydrazone (FCCP) (0.5 µM) and antimycin A/rotenone (1 µM each) (all compound concentrations were optimized to generate the maximum effect in the absence of toxicity). After stress test compound injections there were 3 measurement cycles before the injection of the next stress test compound. This enabled the calculation of basal respiration (OCR prior to oligomycin injection—non-mitochondrial OCR), proton leak (OCR after oligomycin injection—non-mitochondrial OCR), ATP-linked OCR (basal respiration—proton leak—non-mitochondrial OCR), maximal respiration (first injection after FCCP injection—non-mitochondrial OCR) and spare respiratory capacity (maximal respiration—basal respiration) (Figure 2).

##### *In situ* respiratory complex assay in permeabilized cells

Culture medium was replaced with mitochondrial assay solution (MAS) buffer (MgCl_2_; 5 mM, mannitol; 220 mM, sucrose; 70 mM, KH_2_PO_4_; 10 mM, HEPES; 2 mM, EGTA; 1 mM; BSA; 0.4% w/v) and plasma membrane permeabilizer (PMP) (1 nM) containing constituents to uncouple cells and stimulate oxygen consumption via complex I (ADP; 4.6 mM, malic acid; 30 mM, glutamic acid; 22 mM, BSA; 30 µM, PMP; 1 nM, FCCP; 8 µM) (All compound concentrations were optimized to generate the maximum effect in the absence of toxicity) and flutamide or 2-hydroxyflutamide (10–250 µM). PMP is a recombinant form of perfringolysin O, a cholesterol-specific pore-forming reagent which requires a higher threshold level of cholesterol than native perfringolysin O. This enables selective permeabilization of the cell membrane whilst having little or no effect on cholesterol-deficient mitochondrial membranes (Divakaruni *et al.*, 2013; Salabei *et al.*, 2014). Permeabilization of the cell membrane not only permitted mitochondrial access to all of the substrates and inhibitors used, but also depleted the cells of all cytosolic stores, ensuring that mitochondrial electron transport could be specifically driven by the substrates provided. The use of FCCP-treated (uncoupled) cells ensured that any deviations seen were due to perturbations at specific respiratory complexes and not due to inefficiencies in the coupling of OXPHOS.

**FIG. 3:**
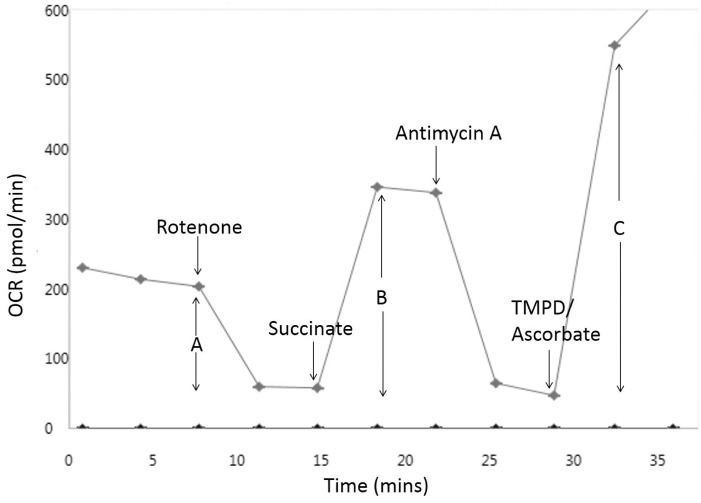
Representative *in situ* respiratory complex assay trace. *In situ* respiratory complex assays consisted of cells in a solution containing substrates for complex I and flutamide/2-hydroxyflutamide or vehicle control (shown) prior to 3 cycles of measurements and a series of compound injections into the cell culture microplate. Injections consisted of rotenone (complex I inhibitor), succinate (complex II substrate), antimycin A (complex III inhibitor), and TMPD/ascorbate (complex IV substrates) with 2 cycles of measurements following each. This series of manipulations enabled the calculation of complex I **(A)**, II **(B),** and IV **(C)** activity. Each measurement cycle was a total of 3 min.

Following a basal measurement of 3 cycles of mix (30 s), wait (30 s), and measure (2 min), sequential injections of A: rotenone (2 µM), B: succinate + rotenone (20 mM, 2 µM, respectively), C: antimycin A (2 µM) and D: ascorbic acid + N,N,N*′*,N*′-*tetramethyl-*p*-phenylenediamine (TMPD) + antimycin A (20 mM, 0.5 mM, and 2 µM, respectively) were performed with a 2 cycle interval between each, allowing measurement of changes in complexes I (A), II (B), and IV (C and D) activity respectively (Figure 3). MAS buffer, all constituents and compound injections were used at pH 7.2.

##### Complex I, II, and III-linked respiration assays in permeabilized cells

Culture medium was replaced with MAS buffer containing constituents to stimulate oxygen consumption via complex I (as previously without FCCP), complex II (ADP; 4.6 mM, succinate; 20 mM, rotenone; 1 µM, BSA; 0.2% w/v, PMP; 1 nM), or complex III (ADP; 4.6 mM, duroquinol; 500 μM, rotenone; 1 μM, malonic acid; 40 μM, BSA; 0.2% w/v, PMP; 1 nM) dependent on the respiratory complex of interest. Following a basal OCR measurement of 3 cycles of mix (30 s), wait (30 s), and measure (2 min), flutamide/2-hydroxyflutamide were injected (10–250 µM) and 3 cycles of measurement made again, prior to a mitochondrial stress test as detailed previously but with changes to stress test compound concentrations; oligomycin (1 μM), FCCP (10 μM), rotenone/antimycin A (2 μM). Changes in complex II activity were also assessed at lower compound concentrations; 2–30 μM (Supplementary Figure S1). Complex I, II, and III activity were defined by the change in complex I, II, or III-stimulated maximal respiration respectively compared with vehicle control.

##### Complex V assay in permeabilized cells

**FIG. 4:**
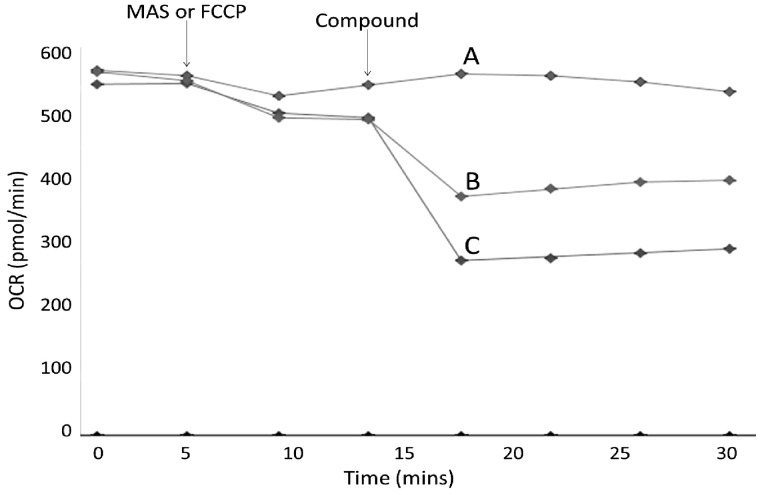
Representative complex V assay trace. Complex V assays consisted of cells in a solution containing substrates for complex IV before a series of compound injections into the cell culture microplate. FCCP (OXPHOS uncoupler) (Trace A) or MAS buffer (Traces B and C) was first injected, followed by 2 cycles of measurements. Flutamide, 2-hydroxyflutamide, oligomycin (positive control), or vehicle control was then injected into both the uncoupled (FCCP-treated) and coupled (MAS-treated) cells. Change in complex V activity was defined as the reduction in OCR of coupled cells upon flutamide/2-hydroxyflutamide injection minus the change in OCR of uncoupled cells, as a % of vehicle control. Injections for traces shown; **A,** FCCP, 250 μM 2-hydroxyflutamide; **B,** MAS, 250 μM 2-hydroxyflutamide; **C,** MAS, Oligomycin. Each measurement cycle was a total of 3 min.

Culture medium was replaced with MAS buffer containing constituents to stimulate oxygen consumption via complex IV as this was not significantly affected by either compound in the *in situ* respiratory complex assay (ADP; 4.6 mM, ascorbic acid; 20 mM, TMPD; 0.5 mM, antimycin A; 2 µM, BSA; 30 µM, PMP; 1 nM). The assay consisted of a basal OCR measurement of 2 cycles of mix (30 s), wait (30 s), and measure (2 min) followed by MAS or FCCP injection (0.5 µM) and 2 measurement cycles. MAS-injected cells remain coupled whereas FCCP-injected cells become uncoupled meaning Complex V (ATP synthase) inhibition should not result in a change in OCR. Either flutamide, 2-hydroxyflutamide (10–250 µM) or oligomycin (positive control; 1 µM) was then injected into both the uncoupled and coupled cells, followed by a final 2 measurement cycles (Figure 4). Change in complex V activity was defined as the difference in flutamide/2-hydroxyflutamide-induced OCR change between coupled (MAS injection) and uncoupled (FCCP injection) cells compared with vehicle control.

##### Normalization of OCR assays to protein content

Following completion of OCR assays, medium was removed from all wells and 20 µl of somatic cell ATP releasing agent was added to each well before shaking for 1 min and transfer of 10 µl of lysate to a 96-well plate and performance of a BCA assay according to the manufacturer’s instructions. Protein content per well was used to normalize OCR.

#### Assessment of Superoxide Level

HepG2 cells were collected by trypsinization and seeded on a 24-well plate (150 000 cells/500 µl/well) and incubated overnight in glucose medium (37**°**C, 5% CO_2_). Cells were then treated with fresh glucose medium containing 15–500 µM flutamide or 2-hydroxyflutamide and incubated for 2 h (37**°**C, 5% CO_2_). mitoSOX red indicator was then added to each well (5 µl/well) and cells were incubated in the dark for 30 min (37**°**C, 5% CO_2_) before collection of cells by trypsinization and absorbance measured at 396/579 nm in a white-walled 96-well plate. Protein content per well was determined using a BCA assay (as described earlier) and was used to normalize superoxide levels.

#### Assessment of NAD^+^/NADH Ratio

HepG2 cells were collected by trypsinization and seeded on a 24-well plate (450 000 cells/500 µl/well) and incubated overnight in glucose medium (37 °C, 5% CO_2_). Cells were then treated with fresh glucose medium containing 30–500 µM flutamide or 2-hydroxyflutamide and incubated for 2 h (37**°**C, 5% CO_2_). NAD^+ ^and NADH concentrations were measured in lysates using a NAD^+^/NADH ratio assay kit according to the manufacturer’s instructions.

#### Data and Statistical Analysis

Experiments were conducted in a minimum of triplicate to ensure the reliability of single values. EC_50_ data were determined by nonlinear regression analysis using GraphPad Prism 5.0. Normality was assessed using a Shapiro-Wilk statistical test. Statistical significance was determined by an unpaired t-test for parametric data and a Mann-Whitney U test for non-parametric data using StatsDirect 2.7.9. *P* < .05 was taken as showing a significant difference.

## RESULTS

### 

#### Flutamide and 2-Hydroxyflutamide Decrease Cellular ATP Content Significantly More in Galactose Medium than Glucose Medium

**FIG. 5:**
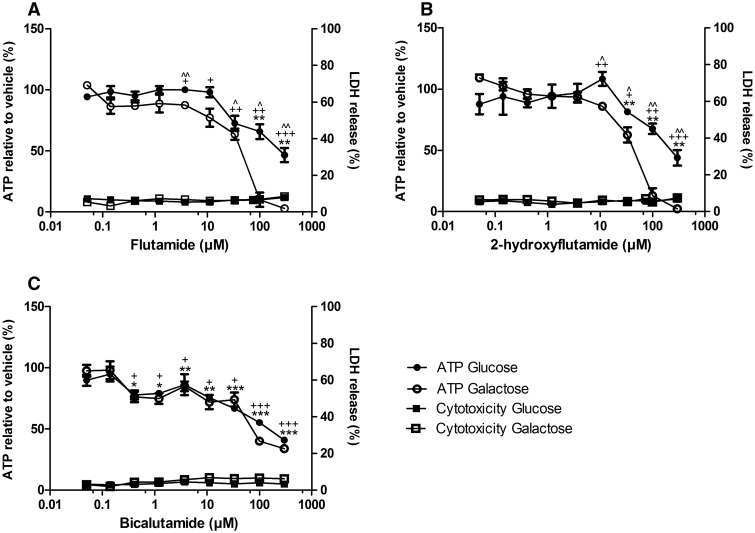
The effect of flutamide **(A)**, 2-hydroxyflutamide **(B)**, and bicalutamide **(C)** exposure on ATP content and cytotoxicity of HepG2 cells (2 h) compared with vehicle control. Serial concentrations of compounds were used up to 300 µM in glucose or galactose media. ATP values are expressed as a percentage of those of the vehicle control, LDH release is expressed as extracellular LDH as a % of total LDH. Statistical significance compared with vehicle control; glucose *P < .05; **P < .01; ***P < .001, galactose ^+^*P* < .05;  ^++^*P* < .01; ^ +++^*P* < .001, between glucose and galactose ^*P* < .05; ^ ^*P* < .01; ^ ^ ^*P* < .001. All results were normalized to µg protein per well. Data are presented as mean ± SEM of n = 3 experiments.

When the concentration of a compound required to reduce cellular ATP content by 50% (EC_50_ATP) is compared in glucose-conditioned vs galactose-conditioned cells, a fold difference of ≥ 2 (EC_50_ATPglu/EC_50_ATPgal ≥ 2) is considered to indicate that the compound contains a mitochondrial liability (Kamalian *et al.*, 2015; Swiss *et al.*, 2013). None of the compounds induced significant cytotoxicity in HepG2 cells (quantified in this study as a significant increase in LDH release) in either glucose or galactose medium. However, the decrease in ATP content induced by flutamide and 2-hydroxyflutamide was significantly more in the galactose medium, but this was not the case for bicalutamide (Figures 5A–C). This translated into an EC_50_ATP ratio (glucose vs galactose media) ≥ 2 for flutamide and 2-hydroxyflutamide, whereas the difference in EC_50_ induced by bicalutamide was ≤ 2 and was not significant (Table 1).

**TABLE 1:** Comparison of the effect of flutamide, 2-hydroxyflutamide, and bicalutamide exposure on intracellular ATP content of HepG2 cells (2 h) in glucose and galactose medium

Compound	EC_50_ATP (μM)		EC_50_ATPglu/EC_50_ATPgal (*P-*value)
	Glucose	Galactose	
Flutamide	255	43.9	5.82 (0.03)
2-hydroxyflutamide	267	48.5	5.51 (0.01)
Bicalutamide	148	103	1.44 (0.22)

EC_50_ATP refers to the concentration of the compound required to reduce cellular ATP content by 50%. Comparison of this value in cells treated in glucose or galactose medium identifies a mitochondrial liability if EC_50_ATPglu/EC_50_ATPgal ≥ 2. *P*-value indicates the significance of EC_50_ATPglu compared with EC_50_ATPgal.

#### Flutamide and 2-Hydroxyflutamide Decrease Mitochondrial Spare Respiratory Capacity and ATP-Linked OCR and Increase Proton Leak

**FIG. 6:**
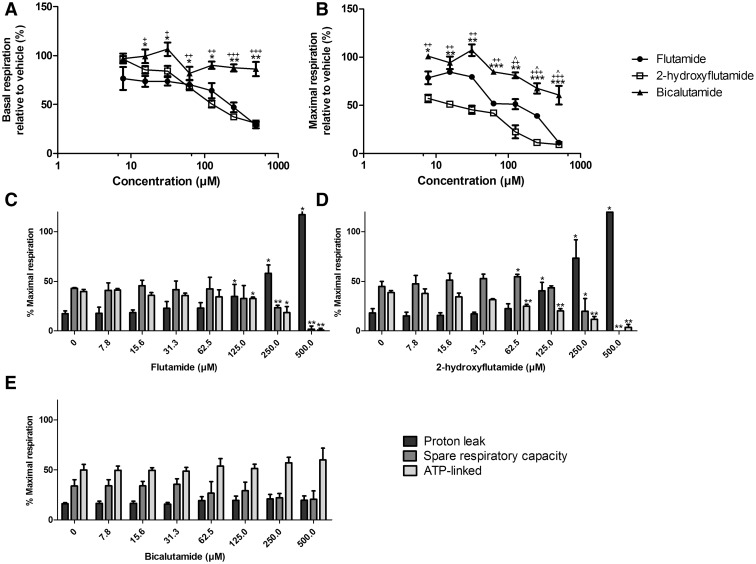
The effect of flutamide, 2-hydroxyflutamide and bicalutamide on mitochondrial OCR in HepG2 cells. Serial concentrations of compounds were used up to 500 µM. **A and B,** Changes in basal and maximal respiration respectively. Changes in proton leak, spare respiratory capacity and ATP-linked OCR induced by flutamide **(C)** 2-hydroxyflutamide **(D)** and bicalutamide **(E)**. Statistical significance compared with vehicle control; (A) and (B) flutamide; *P < .05; ** *P* <.01; *** *P* < .001, 2-hydroxyflutamide; ^+^ *P* <.05;  ^++^ *P* <.01;^ +++^ *P* <.001, and bicalutamide; ^ *P* <.05; ^^ *P* <.01; ^^^ *P* <.001. (C–E) * *P* < .05; ** *P* <.01; *** *P* <.001. All results were normalized to µg protein per well. Data are presented as mean ± SEM of n = 3 experiments.

Following the identification of mitochondrial liabilities in flutamide and 2-hydroxyflutamide, the effect of these compounds on mitochondrial OCR was assessed using a mitochondrial stress test (Figure 2). Both flutamide and 2-hydroxyflutamide induced a significant decrease in basal and maximal respiration whereas bicalutamide only caused a significant decrease in maximal respiration (Figures 6A and B). Both flutamide and 2-hydroxyflutamide (Figures 6C and D) also caused a significant increase in proton leak and a significant decrease in spare respiratory capacity and ATP-linked OCR as a percentage of maximal respiration, changes which were not observed upon bicalutamide-treatment (Figures 6E).

#### 2-Hydroxyflutamide but not Flutamide Induces a Significant Reduction in Complex II Activity

**FIG. 7:**
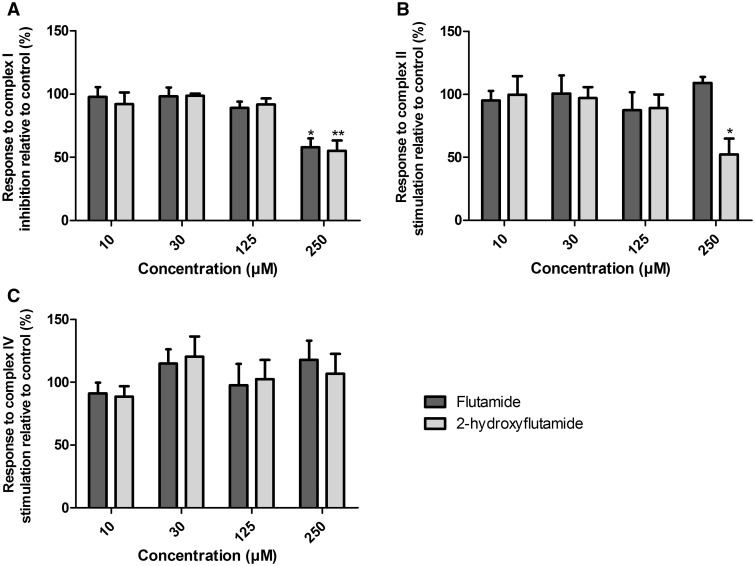
The effect of flutamide and 2-hydroxyflutamide exposure on the activity of mitochondrial respiratory complexes I **(A),** II **(B),** and IV **(C)** in uncoupled, permeabilized HepG2 cells. Compounds were used at 10–250 µM. Statistical significance compared with vehicle control; * *P* < .05; ** *P* <.01; *** *P* <.001. All results were normalized to µg protein per well. Calculation of changes in complex activity is described in Figure 3. Data are presented as mean + SEM of n = 3 experiments.

Given the significant change in mitochondrial OCR induced by flutamide and 2-hydroxyflutamide, the effects of the compounds on the mitochondrial respiratory chain complexes were examined using an *in situ* respiratory complex assay in permeabilized HepG2 cells (Figure 3). Both flutamide and 2-hydroxyflutamide-treated cells responded significantly less to complex I inhibition by rotenone compared with control, implying a significant reduction in complex I activity following treatment with either compound (Figure 7A). Only 2-hydroxyflutamide induced a significantly reduced response to complex II stimulation by succinate, implying compromised complex II activity (Figure 7B). Neither compound appeared to have a significant impact upon complex IV (Figure 7C).

### 2-Hydroxyflutamide Has Additional Mitochondrial Liabilities Compared With Its Parent Compound

**FIG. 8:**
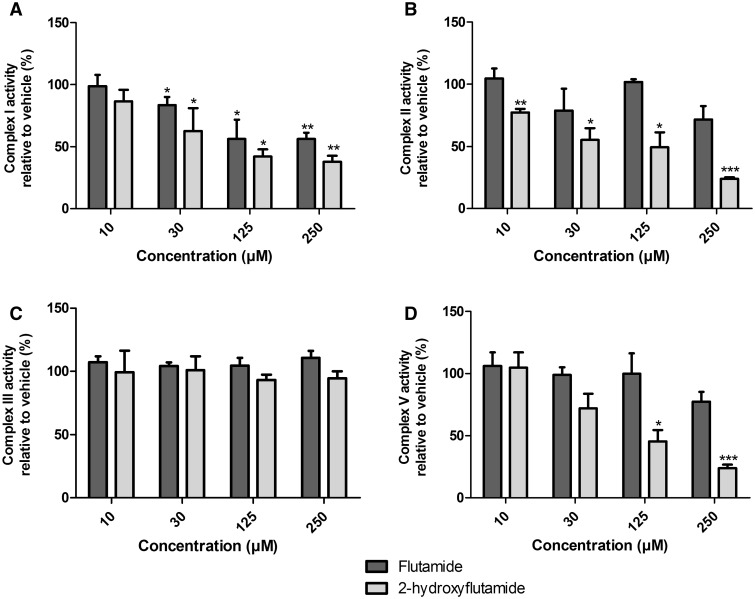
The effect of flutamide and 2-hydroxyflutamide exposure on the activity of mitochondrial respiratory complexes I **(A)**, II **(B)**, III **(C)**, and V **(D)** in permeabilized HepG2 cells. Compounds were used at 10–250 µM. Statistical significance compared with vehicle control; * *P* < .05; ** *P* <.01; *** *P* <.001. Complex I, II, and III activity were defined as complex I, II, or III-stimulated maximal respiration, respectively, compared with vehicle control. Complex V activity was defined as the difference in compound-induced OCR change between coupled and uncoupled cells relative to vehicle control. All results were normalized to µg protein per well. Data are presented as mean + SEM of n = 3 experiments.

Identification of a significant reduction in complex I and II activity following flutamide/2-hydroxyflutamide treatment prompted the specific investigation of compound-induced changes in maximal respiration driven by these complexes. Cells were permeabilized in a solution containing substrates specific for the complex of interest before being treated with flutamide or 2-hydroxyflutamide and a mitochondrial stress test. A significant reduction in complex I activity was induced by both compounds at 30 µM and above (Figure 8A). In agreement with *in situ* respiratory complex assay results, only 2-hydroxyflutamide and not flutamide induced a significant reduction in complex II activity (Figure 8B). Assessment of complex V activity by comparison of compound-induced OCR change in uncoupled and coupled cells showed a significant reduction in complex V activity following treatment with 2-hydroxyflutamide at 125 µM and above, though this was not the case for flutamide (Figure 8D). Assessment of complex III activity showed no significant inhibition induced by either compound (Figure 8C).

#### Flutamide and 2-Hydroxyflutamide Increase Superoxide Levels and Reduce the Intracellular Ratio of NAD+/NADH

**FIG. 9:**
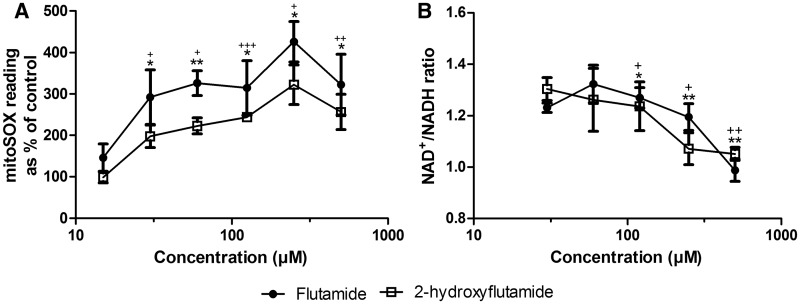
The effect of flutamide and 2-hydroxyflutamide exposure on superoxide levels **(A)** and NAD^+^/NADH ratio **(B)** in HepG2 cells (2 h). Serial concentrations of compounds were used up to 500 µM. Statistical significance compared with vehicle control; flutamide; * *P* <.05; ** *P* <.01; *** *P* <.001, 2-hydroxyflutamide; ^+^ *P* <. 05;^++^ *P* < .01; ^+++^ *P* < .001. mitoSOX results were normalized to µg protein per well. Data are presented as mean ± SEM of n = 3 experiments.

Downstream impacts of mitochondrial dysfunction, particularly complex I inhibition, include increased generation of reactive oxygen species and depletion of cellular NAD^+^. Both flutamide and 2-hydroxyflutamide significantly increased cellular superoxide at 30 µM and above, the same concentration at which significant reductions in complex I-mediated maximal respiration were observed (Figures 8A and 9A). A significant increase in superoxide was also seen in galactose media at the same compound concentrations (Supplementary Figure S2). Both compounds also significantly decreased the NAD^+^/NADH ratio (Figure 9B), though not at concentrations below 100 µM.

Data available from the Dryad Digital Repository (Ball *et al.*, 2016): http://dx.doi.org/10.5061/dryad.jj456

## DISCUSSION

The use of the anti-androgen, flutamide is restricted due to its associated idiosyncratic hepatotoxicity. The rapid first-pass metabolism of flutamide, primarily by CYP1A2 to 2-hydroxyflutamide, results in the parent drug only comprising 2.5% of detectable compound in the plasma following administration (Radwanski *et al.*, 1989). Flutamide is a known inhibitor of mitochondrial respiratory complex I but despite the generation of significant amounts of 2-hydroxyflutamide, the potential mitochondrial liability of this metabolite has not yet been fully examined, prompting its investigation in this study (Coe *et al.*, 2007).

The ability of HepG2 cells in glucose medium to increase glycolysis upon mitochondrial perturbation(s) reduces the ability to identify mitochondrial toxicants and their impact upon cellular ATP content (Crabtree, 1929; Diaz-Ruiz *et al.*, 2011). However, when glucose medium is switched to galactose medium, the net yield of ATP from glycolysis is significantly reduced, increasing the reliance of HepG2 cells upon OXPHOS and mitochondrial toxicity is more evident (Reitzer *et al.*, 1979). This was demonstrated by the significantly greater decrease in cellular ATP content following flutamide or 2-hydroxyflutamide treatment of acutely galactose-conditioned cells vs glucose-cultured cells (Figures 5A and B). An EC_50_ATPglu/EC_50_ATPgal ≥ 2 indicates a mitochondrial liability; a threshold surpassed by flutamide and 2-hydroxyflutamide in the absence of a significant increase in cytotoxicity, suggesting mitochondrial liabilities in both the parent compound and metabolite (Table 1).

The potential for 2-hydroxyflutamide generation in flutamide-treated HepG2 cells must be acknowledged, though the absence of detectable CYP1A2 activity in HepG2 cells and 40-fold lower expression than in primary human hepatocytes, together with the immediate reduction in OCR following flutamide injection during mitochondrial stress tests and complex I respiratory analyses (Supplementary Figure S3) has provided relative confidence that the effect of 2-hydroxyflutamide in flutamide-treated cells would have been negligible during the study (Ranade *et al.*, 2014; Sison-Young *et al.*, 2015).

Given that flutamide has a black box warning for hepatotoxicity, bicalutamide has become one of the front-line treatments for androgen-sensitive prostate cancer. Cells treated with bicalutamide in galactose medium did not have significantly reduced ATP content compared with glucose-conditioned cells, indicating that bicalutamide has no direct mitochondrial liability in HepG2 cells at the concentrations used (Figure 5C).

More detailed analysis of mitochondrial function using OCR analysis and assessment of downstream impacts of mitochondrial perturbations was only performed in glucose media, as in this environment HepG2 cells primarily use OXPHOS for ATP production, meaning that mitochondrial perturbations were still evident whilst deviating less from standard cell culture conditions than with galactose medium (Diaz-Ruiz *et al.*, 2011). OCR analysis using a mitochondrial stress test (Figure 2) demonstrated a significant increase in proton leak and decrease in ATP-linked OCR upon flutamide or 2-hydroxyflutamide treatment (Figures 6C and D), indicative of a loss of electron transport chain function and/or uncoupling of OXPHOS. The spare respiratory capacity of cells also significantly decreased upon treatment with flutamide or 2-hydroxyflutamide, representative of an inability of cells to increase OCR upon treatment with the uncoupler, FCCP (Figures 6C and D). All changes in these parameters were considered relative to maximal respiration, although at high concentrations (500 µM flutamide or 2-hydroxyflutamide) this method was not reliable as the post-FCCP readings suggested that the cells were already uncoupled and resistant to any further dissipation of the proton gradient. These changes were not seen upon bicalutamide treatment at the same concentrations (Figure 6E), with the mitochondria still able to further increase OCR upon FCCP treatment, an adaptive ability which was severely compromised by flutamide or 2-hydroxyflutamide treatment. The fact that bicalutamide did not erode spare respiratory capacity as flutamide or 2-hydroxyflutamide did may contribute to the absence of hepatotoxicity in bicalutamide-treated patients, despite the higher *C*_max_ and longer half-life (1.7 µM and 6 days respectively from a single 50 mg dose) of the active (R)-bicalutamide compared with flutamide (Cockshott, 2004).

To further investigate the mitochondrial target of these compounds, an *in situ* respiratory complex assay was used to identify dysfunction in respiratory complexes I, II and IV using permeabilized cells generated by a cholesterol-specific agent (PMP) (Figure 3). The use of OCR analysis in permeabilized cells as oppose to traditional methods using isolated mitochondria has allowed circumvention of the limitations of mitochondrial isolation, including bias from sub-selection of the mitochondrial population during isolation and limited quality control (Kuznetsov *et al.*, 2008). Neither flutamide nor 2-hydroxyflutamide significantly changed complex IV activity (Figure 7C), prompting individual analysis of only complexes I, II (significant change in activity during *in situ* respiratory complex assays), and III (activity not determined during *in situ* respiratory complex assays) activity. This identified an absence of complex III inhibition by either compound (Figure 8C) but identified both the parent compound and metabolite as inhibitors of complex I activity, though the effect was greater with 2-hydroxyflutamide (Figure 8A). As expected from the *in situ* respiratory complex assay results, only 2-hydroxyflutamide induced a significant decline in complex II activity at 10 μM and above (Figure 8B) (Supplementary Figure S1). This dual inhibition of complex I and II activity by 2-hydroxyflutamide is likely to result in a more severe impact on OXPHOS, as electrons donated by both NADH and FADH_2_ are unable to be accepted. Conversely, if only complex I is inhibited, as in the case of flutamide, then electron entry is still possible downstream via complex II, implying a greater risk associated with 2-hydroxyflutamide. In addition to dual complex I and II liabilities, treatment with 2-hydroxyflutamide also induced a significant decrease in ATP synthase activity at higher concentrations (Figure 8D).

The reduced ratio of NAD^+^/NADH in flutamide and 2-hydroxyflutamide-treated cells was consistent with complex I inhibition by both parent compound and metabolite and suggests the failure of complex I to oxidize NADH (Figure 9B). Imbalances in this ratio have been shown to inhibit NAD^+^-dependent enzymes, particularly sirtuin 3, leading to hyperacetylation of target protein lysine residues and dysregulation of mitochondrial homeostasis (Lombard *et al.*, 2007). The significant increase in superoxide level upon flutamide and 2-hydroxyflutamide exposure occurred at a lower concentration than the significant increase in proton leak (indicative of OXPHOS uncoupling), implying that increased superoxide levels can be attributed to the reduction in complex I activity exhibited in the same concentration range (Figure 9A).

Although the low metabolic capacity of HepG2 cells enabled the study of the parent compound and the selected metabolite separately in this study, the use of a cell line with low metabolism has been limiting in that it has prevented analysis of a wider spectrum of potential metabolites that may be derived from the parent compound and could contribute to the overall toxicity. Specifically, 2-hydroxyflutamide (*C*_max_; 4.4 µM) is the primary metabolite of flutamide, but other minor metabolites are formed, though in much lower abundance, including the hydrolysis product, 3-trifluoromethyl-4-nitroaniline (*C*_max_; 1.31 µM) (Schulz *et al.*, 1988). Another factor which should be considered is the action of flutamide and 2-hydroxyflutamide as inhibitors of taurocholate efflux (IC_50_ 75 and 110 µM, respectively) via bile salt export pump inhibition (Kostrubsky *et al.*, 2007). Importantly, the 16-fold reduction in the expression of this efflux pump in HepG2 cells may have been advantageous as it has allowed direct inhibition of mitochondrial respiratory complexes to be identified in a situation of low active bile-salt export (Sison-Young *et al.*, 2015). This study has therefore indicated that a significant proportion of mitochondrial toxicity could be occurring independently of bile salt-mitochondrial interaction, but the concentrations at which flutamide and 2-hydroxyflutamide have been found to significantly inhibit taurocholate efflux do overlap with the concentrations which significantly inhibit respiratory complex activity, providing scope for a synergistic effect (Aleo *et al.*, 2014).

In conclusion, the rapid generation of 2-hydroxyflutamide *in vivo*, alongside the perturbation of multiple respiratory complexes in HepG2 cells raises the possibility that hepatotoxicity induced by flutamide may be due, at least in part, to the additional mitochondrial liabilities of 2-hydroxyflutamide, the basis of which requires detailed chemical modelling. The generation of this mitotoxic metabolite further differentiates flutamide from bicalutamide in terms of its hepatotoxic potential. The concentrations used in this study do greatly exceed the maximum concentrations of flutamide and 2-hydroxyflutamide seen in patient plasma; however, identification of direct mitochondrial targets of this compound and its metabolite in the absence of bile salt-mediated toxicity has provided a mechanistic starting point for evaluation of interindividual differences in susceptibility to flutamide-induced liver injury. Given the nature of the additional mitochondrial liabilities of 2-hydroxyflutamide, pre-existing functional differences in the basal activity of complexes I, II and V and differential rates of 2-hydroxyflutamide glucuronidation in individuals now form areas of particular interest in the effort to identify susceptible individuals and stratify patients for treatment accordingly.

## SUPPLEMENTARY DATA

Supplementary data are available online at http://toxsci.oxfordjournals.org/.

Supplementary Data
