# Supplementary material for: Identification of the Additional Mitochondrial Liabilities of 2-Hydroxyflutamide When Compared With its Parent Compound, Flutamide in HepG2 Cells
Source: Toxicol Sci. 2016 Jul 13;153(2):341–51. doi: 10.1093/toxsci/kfw126 (PMC5036617; doi:10.1093/toxsci/kfw126)
Supplement: Supplementary Data [file supp_153_2_341__index.html]

Identification of the Additional Mitochondrial Liabilities of 2-Hydroxyflutamide When Compared With its Parent Compound, Flutamide in HepG2 Cells — Identification of the Additional Mitochondrial Liabilities of 2-Hydroxyflutamide When Compared With its Parent Compound, Flutamide in HepG2 Cells — Supplementary Data 

# Identification of the Additional Mitochondrial Liabilities of 2-Hydroxyflutamide When Compared With its Parent Compound, Flutamide in HepG2 Cells

## Supplementary Data

files

- Supplementary Data - pdf file
