# Supplementary material for: Identification of the Additional Mitochondrial Liabilities of 2-Hydroxyflutamide When Compared With its Parent Compound, Flutamide in HepG2 Cells
Source: Toxicol Sci. 2016 Jul 13;153(2):341–51. doi: 10.1093/toxsci/kfw126 (PMC5036617; doi:10.1093/toxsci/kfw126)
Supplement: Supplementary Data [file supp_kfw126_toxsci-16-0110-File011.pdf]

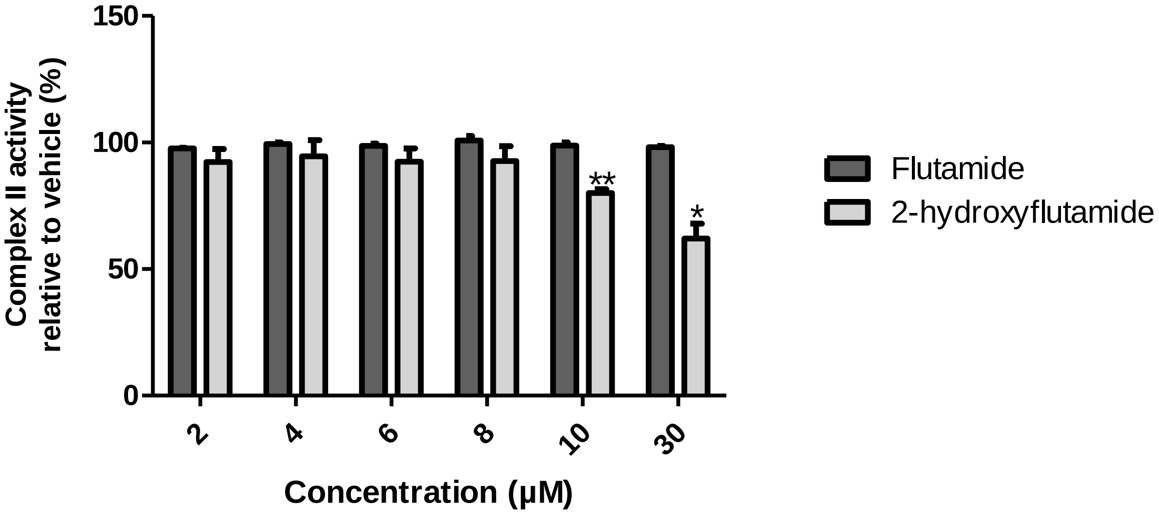

<u>Supplementary Figure 1</u> **The effect of flutamide and 2-hydroxyflutamide exposure on the activity of mitochondrial respiratory complex II in permeabilised HepG2 cells.** Compounds were used at 2-30 µM to include the C_{max} of 2-hydroxyflutamide (4.4 µM). Statistical significance compared to vehicle control; *P<0.05;**P<0.01;***P<0.001. Complex II activity was defined as complex II-stimulated maximal respiration compared to vehicle control. All results were normalised to µg protein per well. Data are presented as mean + SEM of n=3 experiments.

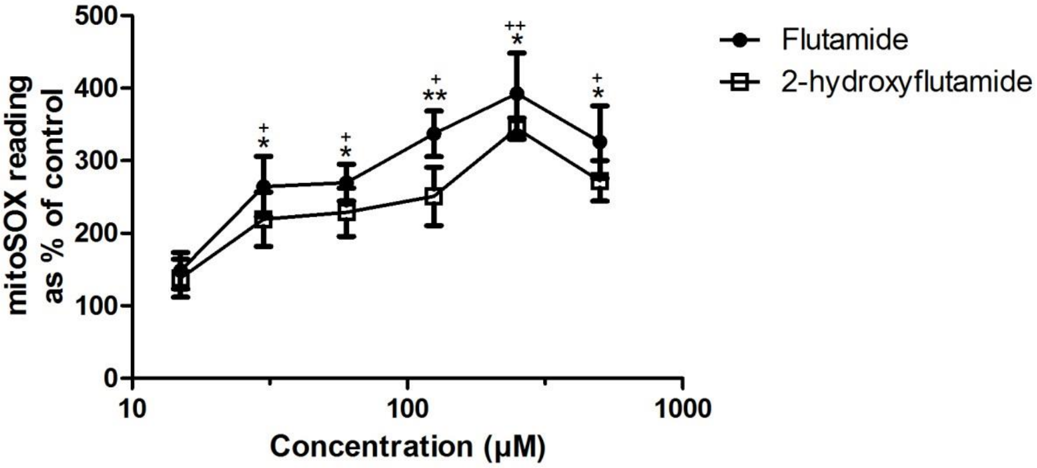

<u>Supplementary Figure 2</u> **The effect of flutamide and 2-hydroxyflutamide exposure on superoxide levels in acutely galactose-conditioned HepG2 cells (2h)**. Serial concentrations of compounds were used up to 500 µM. Statistical significance compared to vehicle control; flutamide; *P<0.05;**P<0.01;***P<0.001, 2-hydroxyflutamide; $^{+}$P<0.05;$^{+}$P<0.01;$^{+++}$P<0.001. All results were normalised to µg protein per well. Data are presented as mean ± SEM of n=3 experiments.

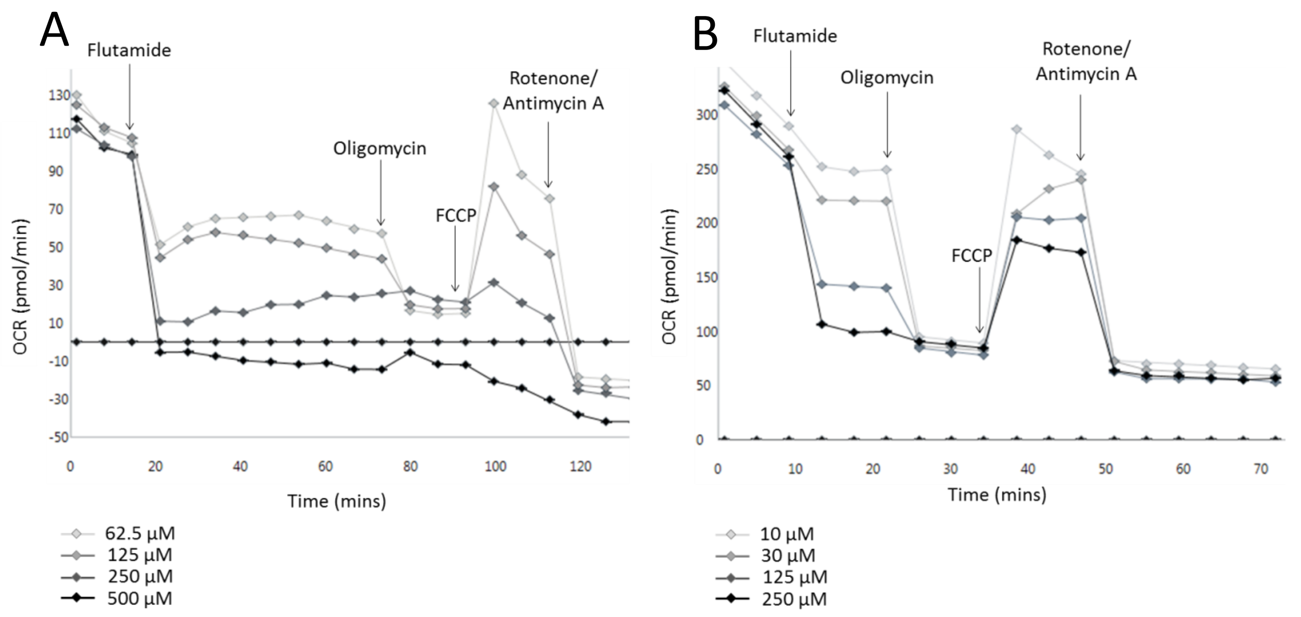

<u>Supplementary Figure 3</u> Representative mitochondrial stress test (A) and complex I assay (B) in flutamide-treated HepG2 cells. Concentrations up to 500 µM (A) or 250 µM (B) of flutamide were used. Mitochondrial stress tests were performed as described in Figure 2. Complex I assays were performed in permeabilised cells in a solution containing substrates required for complex I stimulation, prior to flutamide injection and a mitochondrial stress test. In both assays, upon injection of flutamide there was an immediate concentration-dependent reduction in OCR, supporting the mitochondrial toxicity of flutamide prior to any significant metabolism to 2-hydroxyflutamide.
